# Telemedicine application to headache: a critical review

**DOI:** 10.1007/s10072-022-05910-6

**Published:** 2022-01-24

**Authors:** Emanuele Spina, Gioacchino Tedeschi, Antonio Russo, Francesca Trojsi, Rosa Iodice, Stefano Tozza, Aniello Iovino, Francesco Iodice, Gianmarco Abbadessa, Francesco di Lorenzo, Giuseppina Miele, Elisabetta Maida, Giovanni Cerullo, Maddalena Sparaco, Marcello Silvestro, Letizia Leocani, Simona Bonavita, Fiore Manganelli, Luigi Lavorgna, on behalf of Digital Technologies, Web and Social Media Study Group of the Italian Society of Neurology (SIN)

**Affiliations:** 1 Department of Neuroscience, Reproductive Science and Odontostomatology, University of Naples “Federico II” Via Pansini, 5, 81028 Naples, Italy; 2 Department of Advanced Medical and Surgical Sciences, AOU University of Campania “Luigi Vanvitelli”, Caserta, Italy; 3 1st Clinic Of Neurology, AOU University of Campania “Luigi Vanvitelli,”, Caserta, Italy; 4 IRCSS San Raffaele Pisana, Neurology Unit, Rome, Italy; 5 IRCSS Santa Lucia, Neurology Unit, Rome, Italy; 6 Ospedale del Mare, Napoli, Italy; 7 Experimental Neurophysiology Unit, Institute of Experimental Neurology (INSPE), San Raffaele Scientific Institute, Milan, Italy; 8 Vita-Salute San Raffaele University, Milan, Italy

**Keywords:** Telemedicine, Tele-rehabilitation, Telehealth, Headache, Migraine

## Abstract

**Background:**

Migraine affects more than a billion people all over the world and requires critical employment of healthcare resources. Telemedicine could be a reasonable tool to manage people suffering from headaches, and it received a big push from the COVID-19 pandemic.

**Objective:**

This review aims to propose a practical approach for the virtual management of these patients.

**Methods:**

To do this, we conducted a literature search, including 32 articles relevant to the topic treated in this review.

**Results:**

The most challenging step in telemedicine applied to practical neurology remains the clinical assessment, but through a careful headache history and a recently proposed entirely virtual neurological assessment, this hitch can be easily overcome. Electronic diary compilations and virtual administration of disability-measuring scales, conversely, are the key features of effective long-term follow-up although we do not have apps that met the criteria of scientific reliability. Furthermore, tele-rehabilitation seems to be effective and has demonstrated to be a solution to alternatively treat chronic patients at home, and can be considered part of the remote management of headache patients. Moreover, virtual management of headaches finds an application in specific communities of patients, as pediatric patients and for rural communities of low- and middle-income countries suffer from health disparities, with inadequate resources and knowledge gaps.

**Conclusion:**

Telemedicine could be promising for patients with no regular or convenient access to headache specialists and seems to be a priority in managing migraine patients to avoid non-urgent hospitalizations

## Introduction

Migraine affects more than a billion of people all over the world representing the second disease for years lived with disability, the first one in people under 50 years of age [1] requiring a critical employment of healthcare resources. However, since coronavirus disease 2019 (COVID-19) progressively spread around the world, and, even more since March 11, 2020, when the World Health Organization declared COVID-19 as a pandemic, human and organizational resources—once dedicated to migraine—have been redeployed to COVID-19 management. Therefore, migraine consultations have been cancelled, postponed, or converted into telemedicine. During COVID-19 pandemic, it became mandatory to find alternative strategies for patient management, as telemedicine.

Headache healthcare will occupy an important role in the future of telemedicine because people who suffer from migraine represent approximately the 28% of any neurological outpatient clinic and because headache has so many different patterns of presentation that can lead to the possibility of being pointed out by other professional figures such as internist or gynaecologist, and then addressed to the neurologist [2].

On the other hand, telemedicine is not a novelty for physicians [3–5], although its widespread use has been precluded due to some uncertainties on the real efficacy and safety in comparison to face-to-face visits, to licensing restrictions, and technological and organizational issues [6, 7].

Nevertheless, in the last decade, clinical studies demonstrated that patients perceive telemedicine as cost-effective and worthwhile [8] (especially when using tablet computers as an alternative to traditional telehealth technologies) [9], with satisfaction rates and outcomes similar to traditional in-person visits [10, 11]. Furthermore, with the COVID-19 pandemic, many approaches have been proposed to manage neurological patients with telemedicine; the American Academy of Neurology developed a guidance on technology best practices and regulations [12], specific for the coronavirus emergency, and suggestion on how to perform neurologic examinations remotely. Regarding headache patients, research of the American Headache Society established the need for higher frequency of remote consultations than for other neurological patients, raising the necessity of a correct approach for their virtual management [13].

The primary goal of this paper is to propose an approach based on the available literature to manage headache patients in a virtual way, from the first visit to follow-up and therapeutical management, assuming that technical needs for a correct remote interaction have been properly satisfied.

## Search strategy and selection criteria

A literature search for articles from 1998 to 2021 was conducted in the databases, including PubMed and American Academy of Neurology site using the following Medical Subject Headings (MeSH) terms and key words (also in combination): “headache,” “telemedicine,” “telerehabilitation,” “Covid-19,” “e-health,” and “migraine,” retrieving 139 results.

There were no language restrictions. Relevant articles were identified and located individually in PubMed and American Academy of Neurology site to examine citing and cited-by articles. The final reference list was generated based on relevance to the topics covered in this review; in particular, we decided to include only articles that clearly specified in methods the visit setting, the way to assess and monitoring patients and the possibility of tele-rehabilitation, with a more specific focus on specific communities of headache-patients. We therefore excluded case reports, paper not focusing mainly on headache and those lacking of practical proposals of patient management. Thirty-two studies, answering the aim of this review, were selected and reported.

## First clinical assessment

The most challenging step in telemedicine applied to practical neurology remains the clinical assessment, as neurology is a specialty still highly trusted on semeiotics and where the physical proximity is necessary (e.g. investigation of fundus oculi, reflexes, vestibular system, elicitation and identification of trigger points). In headache patients’ visits, one of the first purposes is to distinguish primary (providing for about 90% of headaches) from secondary headaches that can mimic, or may co-exist along with, a primary headache. The first step is taking a good headache history, to disentangle benign from potentially worrisome headaches. In other terms, a careful anamnesis represents the main diagnostic tool allowing the identification of primary and secondary headaches [12]. The hegemonic role of clinical history in headache medicine is of particular significance overcoming the obvious limitations related to difficulties in performing a complete neurological examination in the telemedicine scenario. To rule out significant secondary causes for headache, neurologists should always keep in mind the “headache red flags”: onset >50 years; sudden onset; headaches increasing in frequency and severity; new-onset headache in a patient with risk factors for HIV infection or cancer; signs of systemic illness; focal neurological signs or symptoms of disease (excluding aura); recent trauma [14].

Available data showed that the virtual approach is safe needing at least 20.000 remote consultations to miss one secondary headache [15]. Moreover, clinical history and specific headache patterns can adequately address neuroimaging investigations (e.g. for patients presenting with a recent-onset of severe non-remitting headache). The second step is the neurological examination, during which obvious difficulties arise, despite its general feasibility with interactive audio and video remote communication between patients and physicians. Recently, Al Hussona et al. proposed a complete virtual neurological assessment [16]. This could be sufficient for headache patients in whom the aim is to exclude gross neurological deficit and, in absence of other red flags, allows physician to proceed with requests for neuroimaging (if needed) and/or therapeutic plan.

A recent survey conducted in Norway showed that neurologists define headache and epilepsy the most suitable neurological diseases for telemedicine management because in both diseases, medical history has a crucial importance in therapeutic decision compared, for example, to movement disorders, in which neurological evaluation usually guide therapeutic decision [17]. The only real limitation declared by author is about performing fundoscopy in headache patients but Evans et al., in the webinar of the American Academy of Neurology for implementing recommendations for Teleneurology during Covid-19, showed some smartphone-based tools to perform remote fundoscopy examination, overcoming this limitation [18]. After collection of medical history, with assessment of the frequency and pattern of headache episodes in order to divide patients in groups of different severity (<4; 4–14; >15 attacks/month and newly diagnosed headache), neurological examination, presence and type of preventive therapies, measurement of functional disability through the Headache Impact Test (HIT)-6, Visual analog Scale (VAS), and Migraine Disability Assessment (MIDAS) [19], the frequency of remote consultations will be established.

## Remote monitoring

In the headache management, follow-up visits and monitoring of therapy response are potentially fully replaceable by implementing mobile health (mHealth) tools. Once assessed the “benign” condition and established a therapeutic plan, it is quite easy to verify the clinical progresses of headache patients. The first step is to encourage patients to note in electronic daily diaries the number and intensities of headache attacks, with duration, site (unilateral or diffuse), associated symptoms (vomiting, nausea, photo/phonophobia), grade of disability (mild, moderate, severe), triggering factors, used drugs (NSAIDS, triptans -, dosage, effectiveness) and, for women, menses. There is growing evidence supporting the role of e-diary; indeed, the use of an electronic diary and the remote monitoring for migraine and medication overuse headache patients have been shown to be superior to classic monitoring strategies (i.e. paper diary), in reducing days with headache, the use of symptomatic drugs, and in increasing adherence to treatment [20]. More recently, headache specialists from the University of Leiden proposed a new e-diary with an automatic algorithm able to identify the type of acute attacks (e.g. migraine or tension-type), number of days with headache with an automatic classification of patients according to the type of headache and severity of the disease [21]. These digital instruments allow neurologists to capture a relatively broad range of information for migraine-tracking purposes and to find, in a small set of data, relevant information for the management of migraine, such as frequency of days with headache, frequency of acute medication intake and functional impairment (the so-called 3 Fs rule) [13]. The main issue in the monitoring of headache patients is about developing mobile apps: although recently many apps have been developed to monitor migraine, the lack of robust scientific data makes their use for medical purposes less practicable [19]. Research conducted up to 2016 identified only six article supporting the use of certain apps (Smartphone Headache Diary, myWHI diary, iHeadache, MyMigraines and MyMigrainesPro, Migraine), pointing out the lack of an optimal remote access to patients’ data, a key property to allow the physician to confirm or modify therapy and, eventually, in case of therapeutic failure, to question the diagnosis [22]. A very recent review evaluated the quality of available headache management apps using behavioral change techniques (BCT), representing specific and evidence-based strategies associated with increased treatment effectiveness through self-monitoring, goal setting, and personalized feedback. Quality assessment of the identified apps took into account patients’ engagement, functionality, aesthetics, and information, and 16 out of 55 ranged from good (4/5) to excellent (5/5). The strength of the BCT-based approach is based on the presence of recognized, evidence-based, strategies to improve headaches and it will surely have a key role in the future management of this class of diseases; nevertheless, none of these apps has been tested in RCTs, so it is not possible to assess with certainty their effectiveness (Table 1) [23]. On the contrary, a strength in remote monitoring of migraine is represented by the scores of the MIDAS and HIT-6 questionnaires, widely used in clinical practice. These scores can be effortlessly transferred to digital platforms, by means of scientifically validated and developed apps, to share strategical information for patients’ management, i.e. discussing adjunctive pharmacological or not-pharmacological preventive measures, or delivering behavioral treatments for migraine patients [24]. Recently, wearable devices have been proposed to passively monitor vital signs with the purpose to verify the presence of some migraine attack predictors or to explore the body answer to headache pain [25].

**Table 1:** Main clinical trials of telemedicine in headache management

Authors	Disease	Design	Tool	Outcome and efficacy	Strength	Limits
Harper et al. [9]	Neurological chronic disease	Observational, prospective	Teleconsultation via tablet technologies	High level of physician satisfaction		Lack of measure of patients satisfaction and clinical outcome
Muller et al. [10]	Non-acute headaches	Observational, randomized, prospective	Videoconference system	High level of patients satisfaction, fewer GPs following visit for headache in TM group	Randomized design	No clinical outcome provided
Muller et al. [15]	Non-acute headaches	Observational, randomized, prospective	Videoconference system	No difference in long-term efficacy between traditional and TM group	Long-term follow-up, inclusion of clinical outcomes	Lack of placebo group and blinding
Tassorelli et al. [20]	Medication Overuse headache	Observational, multicenter, controlled, prospective	Follow-up with electronic diary vs. classic paper diary	Patients in electronic diary group were overuse-free in higher percentage	Design of the study	Not randomized
Van Casteren et al. [21]	Migraine	Observational, prospective	Daily compilation of electronic diary	High efficacy of electronic diary for diagnosis and follow-up of migraine patients	Enabled physician to differentiate between migraine and headache	Not blinded
Sharawat et al. [30]	Pediatric Headache	Observational Prospective	Video and phone call	Teleconsultation is feasible and effective for pediatric headache management	Showed satisfaction also in pediatric population care	Lack of clinical outcome
Adcock et al. [32].	Headache	Observational, prospective	Virtual educational program to local healthcare promoters (Guatemala)	Healthcare promoters reported an increase in subjective knowledge in neuroscience	Developed sustainable methods to train healthcare promoters in rural areas	Lack of objective evidence of improved knowledge
Friedman et al. [34]	Migraine	One-year randomized clinical trial	Zoom video call	TM is not inferior in improving clinical outcomes than traditional approach and is cost and time-saving	TM is a viable method of conducting headache follow-up with patients and physicians satisfaction	Number of not-completed visit

Therefore, web-based survey was an example of asynchronous telemedicine, using to obtain information about some characteristics of migraine patients: for example, a study demonstrated that, during COVID-19 pandemic period, a higher percentage of people with migraine were depressed, even higher than of people with Multiple Sclerosis (50% vs. 43%, *p* = 0.04) [26].

## Tele-rehabilitation approaches

A rehabilitative approach, intended as a non-pharmacological long-time support, together with pharmacological therapy, can significantly improve medical condition of headache patients [25]. Tele-rehabilitation, with both behavioral and physical therapy, seems to be effective and has demonstrated to be a solution to alternatively treat chronic patients at home, but need confirmation from large, controlled clinical trials [25, 27]. As a matter of fact, there are only few applications of tele-rehabilitation to headache suffering patients [27]. Tele-rehabilitation can be considered part of the remote management of headache patients; during the follow-up visits, indeed, physician or paramedic worker should also focus on video assessment of the home environment, on patient education about pharmacotherapies (resulting in a reduction of medication-overuse headaches), lifestyle change, stress management training, cognitive therapy for pain and psychiatric comorbidities [25]. Furthermore, it has been shown that regular daily sessions of mindfulness, held by means of a smartphone application and conducted by an expert physician, have been well-accepted and effective in a cohort of chronic migraine patients with medication overuse headache [28]. Similarly, a recent narrative review pointed out the efficacy of behavioral therapy for migraine patients administered through electronic health (e-health–telecommunication-based health). On the other side, the same review showed the absence of specific mobile app (m-health) to provide these therapies [29]. This novel strategy could be incorporated into the multidisciplinary approach to migraineurs, since it may support and engage patients between outpatient visits and encourage them to play a proactive role in the management of their pain.

## Telemedicine experiences in specific communities

Interestingly, telemedicine is also a convenient option for routine pediatric headache follow-up visits, resulting in high physicians, patient and family satisfaction and cost savings, reporting in 85–90% of cases the willingness to continue the remote consulting beyond the pandemic resolution. Indeed, 90% of children were compliant with medication and 80% with lifestyle modifications without any concerns about safety [30]. Telemedicine options for children suffering from headache demonstrated to be cost-effective, saving up to 600 INR and avoiding long distance travels [31].

Regarding rural population, a recent survey conducted in Norway, apart from pointing out the high level of acceptance and feasibility (except for minor technical issues), hypothesized that telemedicine could become the standard strategy for consultations; the cost saving for each visit has been estimated in about 300 euros with savings up to 8 travelling hours [32]. Furthermore, rural communities of low- and middle-income countries suffer from health disparities, with inadequate resources and knowledge gaps. Trying to equalize these differences, a group of neurologists developed a 1-year project in a rural town of Guatemala: firstly, they identified neurological conditions that required attention through a questionnaire administered to the local population, in which people could report their main neurological complaint; afterwards, neurologists engaged local health promotors in monthly tele-lectures for 1 year, focusing on neurological troubles raised by patients, in order to improve medical assistance. The main issue complained by the local population was headache, in particular migraine, and, at the end of the year, health promotors expressed a high level of satisfaction for this program. At this moment, there are no data about the long-term impact on morbidity and mortality rates; nevertheless, telemedicine can be a way to level the existing disparities between urban and rural sectors of the world [33].

## Conclusion

It has been widely demonstrated that telemedicine is equally effective compared to in-person evaluation and treatment of headache [10]. More recently, improvement in migraine disability for participants in the telemedicine group has been demonstrated not to be different from in-office patients with severe migraine-related disability [34]. Altogether, telemedicine is a feasible mode of management and an effective alternative to in-office visits for follow-up migraine care, allowing a higher physician productivity and a better patient’s access because of its time and cost-effectiveness and convenience. Although controlled trials are still needed to assess the safety and effectiveness of the remote approach, a newly diagnosed patient with headache could be entirely followed up from remote with sufficient confidence.

In conclusion, telemedicine could be promising for patients with no regular or convenient access to headache specialists and seems to be a priority in managing migraine patients to avoid non-urgent hospitalizations at the time of COVID-19 pandemic, where unnecessary physical contact between physicians and patients is highly discouraged.
